# Plantar Sole Unweighting Alters the Sensory Transmission to the Cortical Areas

**DOI:** 10.3389/fnhum.2017.00220

**Published:** 2017-05-10

**Authors:** Laurence Mouchnino, Olivia Lhomond, Clément Morant, Pascale Chavet

**Affiliations:** ^1^Aix-Marseille Université, CNRS, Laboratoire de Neurosciences Cognitives, FR 3CMarseille, France; ^2^Aix-Marseille Université, CNRS, Institut des Sciences du MouvementMarseille, France

**Keywords:** plantar sole afferents, unweighting, EEG, standing balance

## Abstract

It is well established that somatosensory inputs to the cortex undergo an early and a later stage of processing. The later has been shown to be enhanced when the earlier transmission decreased. In this framework, mechanical factors such as the mechanical stress to which sensors are subjected when wearing a loaded vest are associated with a decrease in sensory transmission. This decrease is in turn associated with an increase in the late sensory processes originating from cortical areas. We hypothesized that unweighting the plantar sole should lead to a facilitation of the sensory transmission. To test this hypothesis, we recorded cortical somatosensory evoked potentials (SEPs) of individuals following cutaneous stimulation (by mean of an electrical stimulation of the foot sole) in different conditions of unweighting when standing still with eyes closed. To this end, the effective bodyweight (BW) was reduced from 100% BW to 40% BW. Contrary to what was expected, we found an attenuation of sensory information when the BW was unweighted to 41% which was not compensated by an increase of the late SEP component. Overall these results suggested that the attenuation of sensory transmission observed in 40 BW condition was not solely due to the absence of forces acting on the sole of the feet but rather to the current relevance of the afferent signals related to the balance constraints of the task.

## Introduction

Somatosensory processes have been accorded an important role in triggering and shaping rapid postural responses to unexpected perturbation of the support surface while standing. Indeed, when removing somatosensory inputs in cats with Pirydoxine, Stapley et al. (2002) showed delayed postural responses. The importance of cutaneous inputs in the setting of forces exerted on the ground is supported by a deficit in weight-bearing during locomotion in cats after cutaneous nerve section (Bouyer and Rossignol, 2003). Equally in humans, the significance of cutaneous inputs for controlling postural adjustments has been evidenced by studies of anesthetized foot plantar soles (Do et al., 1990). In addition, in vestibular-loss animals after bilateral labyrinthectomy (Inglis and Macpherson, 1995), the latencies of the postural responses were normal (~375 ms) or even earlier (~325 ms) suggesting a critical role of somatosensory inputs in balance control during perturbation rather than a vestibular-based control. However, when balance control is not challenged (i.e., due to a perturbation or voluntary movements) during the maintenance of normal standing, Meyer et al. (2004) showed that the reduced plantar sensitivity after anesthesia did not alter the postural sway. These studies and others (Ruget et al., 2008; Mouchnino and Blouin, 2013) have highlighted the role of cutaneous afferents when relevant for the task (i.e., challenged balance control). Remarkedly, modulation of the excitability of somatosensory areas can be observed in tasks requiring high somatosensory control (Staines et al., 1997; McIlroy et al., 2003). Indeed, cortical responsiveness to sensory stimuli can be increased in challenging balance situations while standing still (Bolton et al., 2011). For instance, using the somatosensory-evoked potential (SEP) technique, Bolton et al. (2011) found an increased sensitivity to somatosensory inputs of the hand when participants, who were standing with one foot in front of the other (i.e., Romberg’s challenging balance task), lightly touched a fixed support surface with their hand. Importantly, this sensory facilitation was associated with improved balance control (i.e., less postural oscillations) compared to a condition with the same light touch on a support attached to the participant’s wrist (i.e., not referenced to the external environment). Bolton et al. (2011) concluded that the external-referenced touch enhanced the perception of self-generated postural oscillations relative to the external world. Therefore, enhancing the transmission of relevant somatosensory input from the foot sole during challenging balance control, would allow participants to control body sway relative to the external gravity and balance constraints.

However, compensatory postural regulations and functional consequences are load-dependent changes. Carrying extra weight on the body translates into a decreased of the SEP likely indicating a depressed transmission of cutaneous input (Lhomond et al., 2016). Indeed, such variations were observed by Desmedt and Robertson (1977) as early as 55 ms after a tactile electrical stimulation. This early component was interpreted as reflecting the activity of the primary somatosensory cortex (SI; Hari et al., 1984; Hämäläinen et al., 1990). For example, Salinas et al. (2000) showed that the majority of SI neurons in monkeys were phase-locked with the vibratory stimulus. These neurons encoded the stimulus frequency, suggesting a high relationship of SI activity with the incoming sensory inputs. The decrease in the transmission of the afferent cutaneous inflow arising from the periphery to SI could originate from foot deformation resulting from the extra loading. Indeed, it has been reported that obese individuals (Hills et al., 2001) showed higher pressures under the heel, mid-foot and metatarsal regions of the foot compared to normal-weight individuals. Subsequently other studies have observed a greater total plantar force and a greater total contact area (Gravante et al., 2003; Birtane and Tuna, 2004) in obese individuals. A related study by Vela et al. (1998) showed similar observations when normal-weight individuals were loaded with external weights to simulate obesity. Therefore, skin compression where the tactile receptors were embedded could be at the origin of sensory transmission attenuation. For example, under foot loading, the height of the arch of the foot decreases (Bandholm et al., 2008; McPoil et al., 2009) and almost 50% of this change could be accounted for by skin compression (Wright et al., 2012). These behavioral studies together with Lhomond et al.’s (2016) electrophysiological study suggest that the attenuation of the sensory transmission of cutaneous inputs comes from a mechanical origin due to foot sole loading. This phenomenon may be explained by refractoriness in the peripheral nerves themselves (skin receptors firing is already saturated due to load), by depression of synaptic transmission (slowly adapting receptors reduce their input due to adaptation from the foot sole loading), or by alteration of the transmission anywhere along the ascending sensory pathway and within the cortex itself. Therefore if the mechanoreceptors are even partly silenced by the additional weight compressing the skin of the foot sole, the transmission to S1 should be altered.

On the basis of the behavioral and electrophysiological findings reported above, we hypothesized that unweighting the plantar sole should lead to a facilitation of the sensory transmission. To this end, we recorded cortical SEPs following cutaneous stimulation (by mean of an electrical stimulation of the foot sole) in different conditions of unweighting.

## Materials and Methods

**Figure 1:**
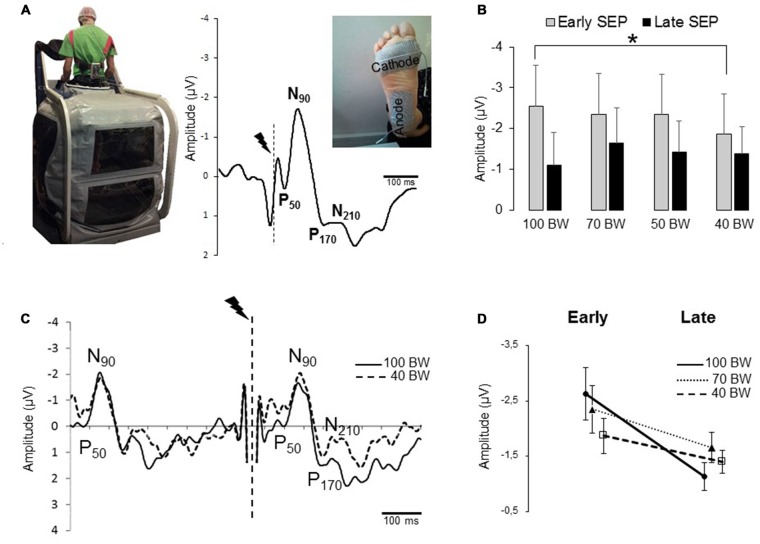
**(A)** Image displaying the position of participants in the Anti-gravity Treadmill^®^. Grand-average EPs for 10 participants recorded over Cz electrode during quiet standing which exhibits an early somatosensory evoked potential (SEP; P_50_-N_90_) followed by a later component (P_170_-N_210_). The vertical dotted lines indicate the stimulation onset. **(B)** Mean for the 80 stimulations of the Early P_50_-N_90_ and late P_170_-N_210_ SEPs amplitudes for all participants (error bars are standard deviation across participants). (*p < 0.05). **(C)** The traces corresponds to the average SEP in 100 bodyweight (BW) and 40 BW conditions for one participant. The break in the curves corresponds to the electrical stimulation artifact. Note that the presence of a first P_50_-N_90_ SEP is due to the time-window shown in the figure that encompasses two stimulations interspace of 500 ms. **(D)** Interaction between the early and late SEPs for 100 BW, 70 and 40 BW conditions. The error bars are standard error of the mean.

Ten participants (6 males and 4 females) performed a bipedal balance task (mean age: 32 ± 13 years; mean height: 173 ± 9 cm; mean weight: 65 ± 4 kg). All participants were free of neurological and musculoskeletal disorders that could influence postural control and had a good fitness base (for review see Paillard, 2017). Informed consent was obtained from all participants, and all procedures were in accord with the ethical standards set out in the Declaration of Helsinki and ethic committee Sud Méditerranée (ID RCB:2010-A00074-35). A Lower Body Positive Pressure (LBPP) treadmill (M310 Anti-gravity Treadmill^®^, AlterG Inc., Fremont, CA, USA) enables an individual’s bodyweight (BW) to be varied. LBPP technology applies a consistent and substantial lifting force opposite to BW. The AlterG^®^ treadmill includes an airtight flexible chamber applied distally to the subject’s iliac crest. This creates local unweighing of the lower limbs while the upper body and all gravity-receptors still experience earth gravity (Sainton et al., 2015; Figure 1A). The electrical signal of the differential pressure (*P*atmospheric − *P*chamber) was recorded with the vertical ground reaction force obtained from four dynamical load cells (XA-shear beam load cell, Sentran^®^, Ontario, CA, USA) located under the frame of the AlterG^®^ treadmill. The ground reaction forces were summed to compute the real BW of the participants.

Participants wore neoprene shorts and stood barefoot on the AlterG^®^ treadmill. Initially, they remained stationary, with their arms alongside their bodies (Figure 1A). The neoprene shorts were sealed to the inflatable chamber. The seal height was adjusted to be level with each participant’s iliac crest, so that the seal itself exerted little or no vertical force. In addition, the compliance properties of the chamber were such that participants’ body was free to move in all directions and participants were even able to walk and run comfortably as shown by Cutuk et al. (2006).

Participants were requested to self-select a side-by-side foot position (approximately feet shoulder-width apart, wide stance) and to keep their eyes closed. Here, particular attention was paid to maintaining the self-selected foot position (i.e., feet shoulder-width apart before each trial) because of the effect of stance width on both postural control and the use of sensory feedback. As shown by Jacobs et al. (2015), the corticomuscular coupling of the bêta frequency band known to represent both afferent and efferent coupling between sensorimotor regions of cerebral cortex and muscle, is sensitive to changes in biomechanical conditions (i.e., wide- or narrow-stance) but not to sensory conditions (foam surface or eyes closed).

The participants were then submitted to different unweighting conditions without changing this initial wide-stance. Four different weighting conditions were applied: 100% of BW, 70 BW, 50 BW and 30 BW. These target values set in the AlterG were held constant for a while (2–3 mn) during the recording session. The changes in BW were applied in a descending sequence (D) from 100D to 30D and then upwards from (U) 50U, 70U and 100U. The participants were blind to the weight conditions and to the sequence. Instead, they were instructed that the BW could be modulated randomly either by increasing or decreasing the weight.

**Figure 2:**
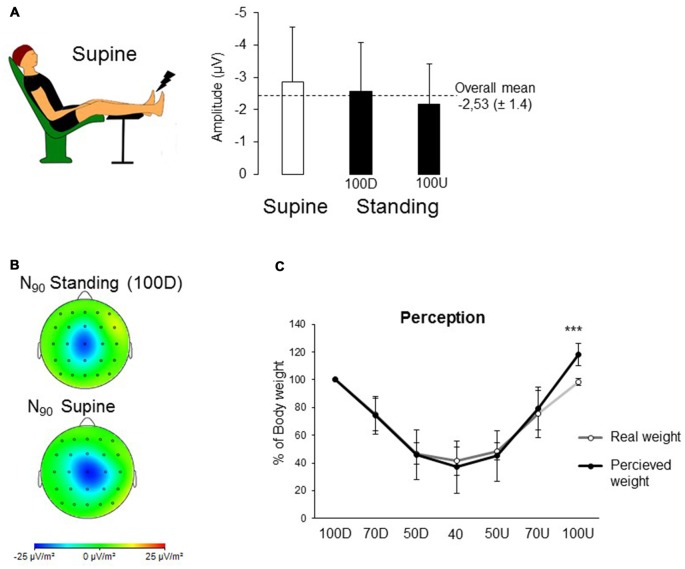
**(A)** Image displaying the supine position. Mean SEPs for the supine position and both 100% of BW conditions. **(B)** To enhance the spatial resolution of the recordings, topographical current source density (CSD) maps were computed using Laplacian transformation with Brain Vision Analyzer. The signal was interpolated with a spherical spline interpolation procedure in order to compute the second order derivatives in two dimensions of space (order of splines: 3; maximal degree of Legendre polynomials: 15). CSDs are independent of the reference electrode site and are much less affected by far-field generators than monopolar recordings. The cortical maps are shown at the latency of the peak negativity (i.e., N90). **(C)** Comparison between the perception of BW given by the participants and the real weight computed by the vertical forces recorded by the treadmill. Each dot corresponds to the mean of 10 participants for all the weighting conditions (error bars are standard deviation across participants; ***p < 0.001).

At every stage of unweighting, the participants were asked to estimate the percentage of BW they were experiencing. In order to avoid any prediction of the percentage of the unweighting, the target weight was not reached directly but only after exploring other weighting. In a control task, participants adopted a semi-supine position (Supine, Figures 1C, 2A) seated in a reclining chair with their plantar soles without a contact with a support surface. The order of the Supine and the Standing task on the treadmill were counterbalanced across participants.

### Stimulation Procedure

While standing the plantar sole of the left foot was stimulated four times with a constant 500 ms interval between each electrical stimulus. This was designed to avoid the “interference phenomenon” (Burke and Gandevia, 1988; i.e., depressed SEPs when stimulations are too close in time, i.e., less than 300 ms according to Morita et al., 1998). The electrical stimulus was delivered by a DS5 isolated bipolar constant current stimulator (Digitimer, Welwyn Garden City, UK). The cathode was located under the metatarsal region and the anode was positioned underneath the heel of the supporting foot (Figure 1A, 5 × 9 cm electrodes, Platinum Foam Electrodes). The stimulation consisted of a single rectangular 10 ms pulse applied under the supporting foot. Taking into account the signature of the cutaneous reflexes reported in Sayenko et al.’s (2009) study, we carefully selected both the position of the electrodes to stimulate the plantar sole as a whole without targeting a specific portion of the foot, and the amplitude of the stimulation to avoid cutaneous reflexes. The stimulation intensity was set as in our previous studies (Mouchnino et al., 2015; Lhomond et al., 2016). For each participant, and while standing, we first found the minimum intensity which gave a constant perception of the stimulations (mean amplitude 6.2 ± 0.1 mA). This stimulation was determined as the baseline value. The stimulation intensity for each participant was set at 25% higher than the baseline value (i.e., well below the motor threshold). Each condition of weighting was divided into 20 standing trials of 5 s. During each trial 4 electrical stimuli were triggered (80 stimulations per condition).

### Electroencephalography and Behavioral Recordings and Analyses

Electroencephalographic (EEG) activity was recorded continuously from 64 Ag/AgCl surface electrodes embedded on an elastic cap (ActiveTwo system, BioSemi, Netherlands). According to the specification of the BioSemi system, “ground” electrodes were replaced by Common Mode Sense active and Driven Right Leg passive electrodes. These two electrodes, located near Pz and POz electrodes, form a feedback loop, which drives the average potential of the participant (the Common Mode voltage) as close as possible to the anolog-digital converter (ADC) reference voltage in the AD-box. The signals were pre-amplified at the electrode sites and post-amplified with DC amplifiers and digitized at a sampling rate of 1024 Hz (24-bit resolution). Signals from each channel were referenced using the average of the 64 scalp electrodes. The signals were further filtered off-line with 35 Hz (high cut-off) filters (digital filters, 48 dB/octave) and 0.1 Hz (low cut-off) filters (digital filters, 12 dB/octave; BrainVision Analyzer 2, Brain Products, Germany).

SEPs, (Figure 1A) were obtained by averaging, for each participant and condition, all synchronized epochs (i.e., 80) relative to the electrical stimulus. The average amplitude of the (−100; −50 ms) pre-stimulus epoch served as baseline. The −50 ms relative to the stimulation was chosen to avoid any artifact related to the stimulation procedure. We examined the SEPs over the Cz electrode as this electrode overlays the sensorimotor cortices on the homunculus, the feet are located on the inner surface of the longitudinal fissure. The earliest discernible positive (P_50_) and negative (N_90_) peaks after each stimulus were identified. Such peaks latencies are comparable to latencies observed by Altenmüller et al. (1995) and Duysens et al. (1995) evoked by stimulating the sural nerve. The fact that the sural nerve is a primarily/exclusively cutaneous nerve (Burke et al., 1981) lends argument for the P_50_-N_90_ originating from cutaneous input. The amplitude of the P_50_-N_90_ waveform was measured peak-to-peak., a late SEP component (P_170_-N_210_) was observed at a latency similar to latencies observed in Lhomond et al.’s (2016) study.

**Figure 3:**
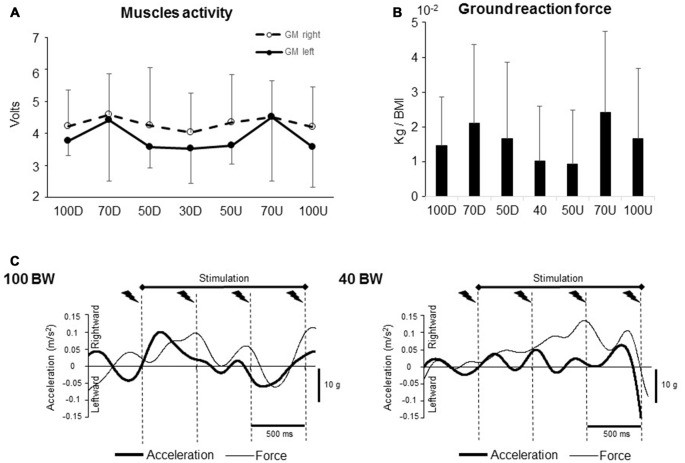
**(A)** Integrals of both right and left gastrocnemius medialis (GM) muscles activity recorded during a 450 ms duration period. **(B)** Integrals of the vertical ground reaction force during a 2100 ms duration period that encompassed the whole stimulation procedure. **(C)** Mean head lateral acceleration and ground reaction force non-rectified traces for one participants (100 BW and 40 BW).

Head acceleration was measured by using a triaxial accelerometer (Model 4630: Measurement Specialties, Virginia, VA, USA) placed on the chin. The rationale for using head acceleration as an index for whole body stability relative to space is that for balance and posture the whole body can be assumed to act as a rigid segment (inverted pendulum model) about the subtalar joint of the feet (MacKinnon and Winter, 1993). For example, Jeka et al. (1997) showed that during a light finger touch on a stationary bar, the lateral displacements of head and center of pressure were in phase and superimposable. For each trial, after applying a 4th order *Butterworth* filter with 3 Hz cut-off frequency on the raw data over time, de-biasing and rectifying the signal, we computed the integral of a 1600 ms time-window which encompassed the four stimulations periods including the P_50_N_90_ component following the last stimulation (Figure 3A).

We analyzed the ground reaction force from one gauge (located on the right non-stimulated side). After applying a 4th order *Butterworth* filter with 3 Hz cut-off frequency on the raw data over time, the data were rectified, integrated and normalized relative to the body mass index of each participant.

Bipolar surface electromyography (EMG; Bortec AMT-8 system; Bortec Biomedical, Calgary, Canada) was used to record bilaterally the activity of the tibialis anterior (TA) and gastrocnemius medialis (GM) muscle. EMG signals were preamplified at the skin site (×1000), analog filtered with a preset bandpass (20–250 Hz) and sampled at 1000 Hz, then rectified. These recordings were performed to evaluate the level of muscle activation during the standing task. To quantify these activations, we computed the integral of the EMG activity (iEMG) for each muscle during five 400 ms time-windows. The first time-window was computed before the stimulation (i.e., baseline [−450; −50]). The other time-windows were computed after each stimulation (i.e., [50; 450], [550; 950], [1050; 1450], [1550; 1950]). As no differences were observed between the four iEMG-windows during the stimulation period we computed the mean iEMG from the four time-windows. One of the participants had no available recordings for the 100D condition and was discarded from the analyses.

### Statistical Analyses

The amplitudes and latencies of the SEPs were submitted to repeated-measures analysis of variance (ANOVA) designed with conditions of weighting (100D, 70D, 50D and 30). Significant effects were further analyzed with Newman-Keuls *post hoc* tests. For the size effect calculation we used the *η*^2^ (Eta squared), and to work out effect size we used the Cohen’s (1988) guidelines (Fritz et al., 2012). We also conducted paired *t*-tests when necessary. The level of significance was set at 5% for all analyses. All dependent variables (EEG and behavioral data) showed normal distributions (i.e., *p* > 0.05, Kolmogorov-Smirnov test).

## Results

The assessment of the precision of the BW level was performed a posteriori; 70 BW corresponded to 75 ± 10%, 50 BW was 47 ± 5% and 30 BW was 41 ± 10% for all participants. In order to determine if the real unweighting experienced by each participant corresponded to the target unweighting set in the AlterG, the real unweighting was compared to a standard value (i.e., target unweighting) for each condition. These analyses revealed that the real weight for the 70 and 50 BW conditions were not different from their standard values (*t*_9_ = 1.32; *p* = 0.21 and *t*_9_ = −1.48; *p* = 0.17, respectively). In the 30 BW condition the real weight is increased relative to the standard value set in the AlterG (*t*_9_ = 3.81; *p* = 0.004). We therefore chose a new and more appropriate standard value of 40 (*t*_9_ = 0.72; *p* = 0.48). For clarity of purpose, we replaced the 30 BW condition by 40 BW below in the results section to denote each unweighting levels.

### Somatosensory Evoked Potentials

During quiet standing, the foot stimulation evoked typical EEG signals. Figure 1A shows the grand average at electrode Cz for all participants. Both an early and a late sensory processes were identified. The early SEP consisted in a small positive component (P_50_) followed by a prominent negative deflection (N_90_). First of all, to assess that decreasing and increasing the weight on the feet (i.e., order effect) did not change the amplitude of the SEP, we compared the 100, 70, 50 Down with the 50, 70, 100 Up. SEP amplitudes were submitted to 2 modes (decreasing, increasing) × 3 BW (100, 70, 50 BW) repeated measures ANOVAs. The results showed that the amplitude of the P_50_-N_90_ SEP did not depend on the order (i.e., Down or Up) of the unweighting (*F*_(1,9)_ = 0.34; *p* = 0.57) nor on the BW (*F*_(2,18)_ = 0.14; *p* = 0.86). Therefore we will use the descending order to compare the 100D, 70D, 50D and 40 BW conditions.

SEP data (amplitude and latencies) were submitted to repeated measures ANOVA with different condition of BW (100D, 70D, 50D and 40) as the main factor. The results showed a BW main effect on the P_50_-N_90_ SEP amplitude (*F*_(3,27)_ = 3.41; *p* = 0.031) with a large size effect of 0.27. As the decrease at 40% BW is relatively small with large standard deviation, we used the Tukey’s HSD test (i.e., less liberal test than the Newman-Keul’s *post hoc* test) and found that the SEP had a smaller amplitude in the 40 BW condition (−1.86 ± 1 μV) compared to the 100 BW conditions (−2.56 ± 1.5 μV; *p* = 0.02; Figure 1B). In addition, no BW effect was observed for the latencies of P_50_ (*F*_(3,27)_ = 0.93; *p* = 0.43; overall mean of 64 ± 17 ms) and of N_90_ (*F*_(3,27)_ = 0.59; *p* = 0.62; overall mean of 96 ± 19 ms). It was noticed that the ANOVA did not show a general BW effect (100, 70, 50 and 40 BW) on the late SEP component (Figure 1B, *F*_(2,27)_ = 1.60; *p* = 0.21).

To assess whether the decreased SEP observed in 40 BW was due to an altered use of mechanoreceptors provoked by the unloading of BW, a supine position (i.e., weightless) was compared to both Standing conditions (i.e., 100D and 100U, Figures 2A,B). The results did not show a condition effect on the early SEP amplitude (overall mean: −2.53 ± 1.4; *F*_(2,18)_ = 2.94; *p* = 0.07) or on the P_50_ and N_90_ latencies (P_50_ overall mean: 62 ms ± 13; *F*_(2,18)_ = 0.60; *p* = 0.55 and N_90_ overall mean: 92 ms ± 15; *F*_(2,18)_ = 0.42; *p* = 0.66).

To further test whether the attenuated transmission of sensory inputs (P_50_-N_90_ SEP) in the 40 BW condition was associated with an altered late potential (P_170_-N_210_ SEP), SEP data were submitted to repeated measures ANOVA with conditions (100 BW, 70 BW and 40 BW) and SEPs components (early P_50_-N_90_ and late P_170_-N_210_ components) as the main factor (Figures 1C,D). We have discarded the 50 BW condition from the analyses to lessen the variability. The results revealed a main component effect (*F*_(1,9)_ = 7.17; *p* = 0.02 with an interaction SEP (early and late components) × BW (*F*_(2,18)_ = 5.09; *p* = 0.17). Post hoc analyses showed that the early components were greater than the late components in 100 BW and 70 BW conditions (*p* < 0.05) and of approximately equal amplitudes in 40 BW condition (*p* = 0.12). In addition *post hoc* analyses confirmed that the early SEP recorded in the 100 BW condition was greater than the early SEP of the 40 BW condition (*p* = 0.016) but not different from the 70 BW condition (*p* = 0.35). What is informative (Hsu, 1996) is that the late SEP in the 70 BW condition was greater than in the 100 BW although this statistical value fell short of the conventional 0.05 cut-off value for statistical significance (*p* = 0.07). No difference was observed between the late SEP in 100 BW and in 40 BW (*p* = 0.23).

### Perception and Behavior

Due to the difference between target value and real weight, it was considered more pertinent to compare the perception of the weight to the real weight. In addition, the 100D condition was excluded as it started the experiment in the AlterG and all participants were aware of the 100% BW condition. With regard to perception (Figure 2C), results showed a significant interaction between conscious perception of the BW and real conditions of weighting (*F*_(5,45)_ = 2.58; *p* = 0.038) with a large size effect of 0.22; *post hoc* analyses confirmed that the participants’ own BW was perceived heavier in the 100 Up (117 ± 8% of BW) than the real BW (i.e., 98 ± 2 of BW; *p* < 0.001).

The behavioral data (activity of ankle musculature, vertical ground reaction force, and head acceleration) were submitted to repeated measures ANOVA with different condition of BW (100D, 70D, 50D 40, 50U, 70U, 100U) as the main factor during the stimulation procedure (i.e., stimulation).

To verify if the difference in the SEPs amplitude was not due to a difference in the motor activity, we compared the iEMG of TA and GM muscles of both legs computed in the different conditions. The muscle activity did not change across the unweighting condition; however, the activity of the left GM showed a slight rise in activity for both 70D and 70U BW conditions without reaching the significant level (Figure 3A, *F*_(6,48)_ = 2.09; *p* = 0.07). No condition effect was observed for the other ankle muscles (*F*_(6,48)_ = 0.38; *p* = 0.88; *F*_(6,48)_ = 1.36; *p* = 0.24; *F*_(6,48)_ = 0.51; *p* = 0.79 for the right GM and right and left TA, respectively). Overall, these results suggest that the depression of the early SEP in 40 BW condition was not related to an increase in muscular activity which indeed could have induced a sensory suppression (Cohen and Starr, 1987; Seki and Fetz, 2012).

After normalization to the body mass index (including participant’s weight and height), ground reaction force and head acceleration data were analyzed (Figure 3C). No difference was observed neither for the forces (Figure 3B, *F*_(6,54)_ = 1.68; *p* = 0.14, for the main condition effect) or for the head acceleration (*F*_(6,54)_ = 1.45; *p* = 0.21, *F*_(6,54)_ = 1.63; *p* = 0.15, in the mediolateral and anteroposterior direction, respectively).

## Discussion

The aim of this study was to identify whether the sensory transmission from the plantar sole tactile receptors in a bipedal standing position is modulated relative to the force acting on the foot sole. A facilitation of the sensory transmission was expected in the unweighting condition.

Surprisingly our results did not show an increase in transmission as expected but rather a decreased early activity over SI in the unweighting 40 BW condition compared to full BW (i.e., 100 BW). One possible explanation for these findings is that the unweighting 40 BW condition with reduced loading of the feet could have induced a change in sensory noise (i.e., background sensory traffic). Indeed, mechanoreceptors adaptation to the static pressure due to normal BW could not take place under such unloading and may give raise to a sensorial “noise” (Weerakkody et al., 2007). This sensorial “noise” or interference phenomenon (Burke and Gandevia, 1988) could be at the origin of a low perception. For example Mildren and Bent (2016) have shown that cutaneous stimulation at different skin regions across the foot can influence proprioception at the ankle joint (i.e., perception of feet orientation). The authors concluded that inputs from cutaneous mechanoreceptors had an influence on ankle proprioception and this error of perception could be due to an inhibition of cutaneous or spindle proprioceptive feedback, causing the perception of smaller movement magnitudes. This sensorial “noise” could be also observed when wearing a loaded vest (i.e., low SEP, Lhomond et al., 2016) or when comparing standing to sitting (Mildren et al., 2016). However, the perception of participants’ weight in the 40 BW condition was preserved (i.e., no difference between the real, 41% and the perceived weight, 37%) despite the decrease sensory transmission (i.e., lower early SEP). The accurate perception of BW in the 40 BW condition could not dismiss the sensory “noise” hypothesis. Indeed, Bays and Wolpert (2007) suggested that the noise in the sensory system could lead to a reweighting of the available sensory sources. Therefore, the integration of other modalities could compensate for the sensory “noise” and preserve an accurate perception of the BW.

While in most previous studies an increase in sensory transmission has been shown to be related to an increased perception of tactile stimuli when relevant to the motor task (Duysens et al., 1995; Cybulska-Klosowicz et al., 2011), our results suggest a less straightforward causal relationship between transmission and perception. In the current study, the perception was altered (i.e., overestimated, about 120%) with the presence of a full amplitude SEP (i.e., 100Up BW) and, conversely perception was preserved with a decreases SEP amplitude (i.e., in 40 BW). Therefore perception does not depend solely on early sensory transmission but rather relies predominantly on processing signals originating from sensorimotor-related neural mechanisms. Among these sensorimotor mechanisms were those involved in the prediction of the sensory consequence of our own action even if this action consists in preserving body equilibrium (Blakemore et al., 1998, 1999a,b; Voisin et al., 2011; Cullen and Brooks, 2015; Benazet et al., 2016).

Evidence for task-specific gating of the cortical transmission in the 40 BW condition observed in the current study parallels that seen in McIlroy et al.’s (2003) study. For instance, these authors showed that the SEPs evoked by tibial nerve stimuli in a seated task while the participants were to relax (i.e., Supine condition here, in our study) were similar to those of a task (termed “Threatened balance”) in which the seated participants were maintaining the position of an inverted pendulum with threat of external perturbation by balancing a platform under their feet (i.e., Standing 100% BW here, in our study). In addition, in a third sitting task without a threat to the stability of the pendulum (i.e., No balance constraints) but with the same forces exerted on the foot sole (i.e., muscle contraction or ankle angle), the SEP was depressed by 28%. This study (McIlroy et al., 2003) together with the depressed SEP in the 40 BW condition (i.e., low balance constraints) may support the idea that the central nervous system decreases sensory transmission according to the decrease in the balance constraints of the task. Indeed, the 40 BW condition did not endanger the equilibrium as it was reported by Ritzmann et al. (2015) in underloading situations during parabolic flight (i.e., 0.16 and 0.38 g). These authors showed that the center of gravity is suitably adjusted above the base of support and that was achieved by a slow body motion control resulting from the noticeably reduced ankle joint torque.

Our results suggest that the brain exerts a dynamic control over the transmission of the afferent signal (i.e., attenuation) according to their current relevance to the task. The idea that the attenuation probably occurs at a cortical level has been previously suggested by Applegate et al. (1988). This study suggests that the attenuation of short latency cerebral potentials during standing relative to voluntary isometric plantarflexion while sitting may not be explicable entirely by the change in background muscle activity and by non-specific effects exerted on relay nuclei by standing because the subcortical component (P_32_-N_38_) was not reduced by stance. Additional support suggesting that the altered transmission of afferent inputs is centrally-driven comes from the late SEP analyses. Remarkably, the decrease of the early SEP in 40 BW condition was not associated with an increase in the late sensory processes (i.e., same amplitude of late SEP in 40 BW and in 100 BW) contrary to what was observed in overloading condition (Lhomond et al., 2016). In this previous study, the enhancement of the late-stage sensory integration was interpreted as a mechanism aimed at compensating for decreased early sensory transmission in order to control whole body stability which was decreased with additional loading. Even though the transmission of cutaneous input is depressed and the late integrative process remained unchanged in the current study, both head acceleration and vertical force exerted onto the ground were similar to the normal weight condition. These results suggested that there was no need for further compensation (i.e., increase late sensory process) as body balance was not endangered by the unweighting 40 BW condition.

In addition, the late SEP was greater and associated with an increased lateral head acceleration during the first unweighting change experienced by the participants (i.e., 70D condition). This condition separates for the first time the gravitational somatosensory information (i.e., altered) provided by the contact forces of the feet with the supporting surface from the vestibular cues provided by the gravity acceleration (i.e., unchanged). The late sensory upregulation together with the decreased whole body stability observed here most likely reflects an enhancement of the integration of somatosensory and vestibular inputs from the head acceleration, to reset an internal model of gravity (Papaxanthis et al., 2003; Indovina et al., 2005; Herold et al., 2017). A similar increased activation has been reported by Miyai et al. (2006) in healthy participants during gait on a treadmill with unusual partial BW support (10%).

In conclusion, our study is the first to examine the unweighting effects on the transmission of afferent inputs from the periphery to the cortical areas during upright standing. We observed a suppression of sensory transmission in particular within a threshold range from 47% to 41% of BW (i.e., respectively, 50 BW and 40 BW conditions) experienced by the healthy participants. This is partly because the tactile information from the foot sole is less relevant in terms of balance constraints with underloading. In this context, as the AlterG^®^ treadmill can be considered as a safety device for loading and unloading lower extremities in patients with lower limb injuries and disorders, the efficacy of the rehabilitation programs should consider the sensory mechanisms together with the motor aspects of standing, walking and running.

## Author Contributions

LM, OL and PC contributed to the conception and design of the work. LM, OL, CM and PC contributed to the acquisition, analysis, or interpretation of data for the work, contributed to the writting of the work or revising it critically.

## Conflict of Interest Statement

The authors declare that the research was conducted in the absence of any commercial or financial relationships that could be construed as a potential conflict of interest.
